# A Case of an Enormous Tumour in the Cavity of the Abdomen

**Published:** 1810-08

**Authors:** Robert Kinglake


### A Case of an enormous Tumour in the Cavity of the Abdomen; by Robert Kinglake, M.D.

It has lately occurred to me to see, in the case of a child about three years of age, a large substantial tumour, situated in the epigastric region, and extending its volume throughout the greater part of the cavity of the abdomen. It appeared to originate from the inferior surface of the small lobe of the liver, at least, it was firmly attached to that part. Its weight was upwards of eight pounds, and its texture was for the most part steatomatous; some portions of it were follicular, and others of various and unequal degrees of hardness. The basis of the tumour was so narrow as to admit of its body being easily moved from side to side when pressed on, which afforded an undulating feel not unlike the fluctuating movement of a fluid. Several medical practitioners supposed it to be a clear case of ascital dropsy, and the patient was referred to me for my opinion as to the propriety of discharging the contained fluid by tapping. Nothing but the irretrievably lost state of the child, from the evidently enlarged size of the liver, and its emaciated and dying state, induced me to advise desisting from the operation, lest it should expedite the death of the little sufferer. In about three days after the child died, when, on opening the cavity of the abdomen, the tumour here described presented itself. It did not appear that there was any accumulation of water in the cavity of the belly.

Had the trocar been plunged into the swelling, as proposed, the deception would have been embarrassing, and would, no doubt, have occasioned additional distress to the patient.

The circumstance of there being a palpable enlargement of the liver, suggested more caution than would otherwise have been observed in treating this case as a mere dropsical affection. When visceral disease unquestionably exists, it deserves to be maturely considered in what proportion a substantial enlargement of organic parts may contribute to that bulk, which should induce suitable doubt and hesitation in concluding it to be absolutely of a fluid nature.

I remember attending a consultation on a case of supposed hydrocele, somewhat similar with respect to the nature of the tumour to the instance here recited, in the Hospital at Gottingen, under the care of Dr. Richter. The swelling was extremely bulky; on examination, it felt uniformly soft and painfully tensive. The question was, whether it was formed by an enlargement of the testicle, or an accumulation of water between its investing coats. The latter opinion predominated, and a trocar was immediately passed into it, when nothing more than a few drops of blood were returned through the canula. The consequent pain became excruciating, and the patient died within eight and forty hours. On examination after death, this ambiguous tumour proved to be a case of sarcocele, and the unsuitable treatment at least hastened the destruction of the patient.

Instances of tumefactions in visceral cavities are perhaps not frequent,* though it is clear that the accretion of

* Bonetus, in his Sepulchretum sive Anatomia Practica, has indeed recorded, among other somewhat analogous instances, the following more particularly striking examples of this disease.

"Scotus dolebat in hypochondriis, lumbis, &c. Supervenit tumor circa costas nothas sinistri lateris ad os xiphoides prominens: Induruerant pancreas et mesenterium, et in molem supra modum latam, longam et crassam excreverant.

"Matronæ aderat tumor durus ad tactum circa mucronatam cartilaginem, renitens, oblongus: Gibba pars hepatis videbatur tanquam novum corpus simæ superstratum, a qua dependebat tumor oblongus instar pyri.

"Vidi anno 1593, in quadam virgine juvene totum abdomen a Liene occupatum, ipsumque ita exuberantem, ut videtur hæc virgo hydrope laborare: Imo quod gravissimi medici in aspectu cadaveris istius mulieris dissecti, se deceptos fuisse fassi sunt: Erat molli abdomine et suffocata, quamadmodum hydrope laborantibus accidit, de vita discessit." Vide Lib. 3, Tit. de Ventris Tumore.

of new substances, under the stimulus of morbid irritation, may happen to almost any extent compatible with life. If an unusual degree of excitement should invite a proportionate supply of nutritive matter, it may be so applied and arranged as ultimately to produce excrescenecs of as large dimensions as those of the tumour which occurred in the case under consideration. That these diseased secretions often happen is very probable, and they will prove more or less destructive, according to the situation they may occupy, and to the nature of the neighbouring parts on which they may injuriously press. Nor is it easy either to know the existence of such tumours, or if known, effectually to counteract and prevent their progressive augmentation. It is, however, useful to be aware of the possibility of such states of disease, that when they actually occur, they may not be mistaken for others, the nature and appropriate treatment of which may be essentially different.

*Taunton, June 12, 1810.*

